# Optoelectronic Properties of Shallow Donor Atom in 2D-Curved Nanostructures Under External Electric and Magnetic Fields

**DOI:** 10.3390/nano15010015

**Published:** 2024-12-26

**Authors:** Soufiane Chouef, Mohammed Hbibi, Reda Boussetta, Abdelaziz El Moussaouy, Farid Falyouni, Omar Mommadi, Carlos Alberto Duque

**Affiliations:** 1 OAPM Group, Laboratory of Materials, Waves, Energy and Environment, Department of Physics, Faculty of Sciences, University Mohamed I, Oujda 60000, Morocco; soufiane.chouef@ump.ac.ma (S.C.); mohamedhbibi093@gmail.com (M.H.); azize10@yahoo.fr (A.E.M.); falyouni@yahoo.fr (F.F.); 2 MEGCE Group, Laboratory of Materials, Waves, Energy and Environment, Department of Physics, Faculty of Sciences, University Mohamed I, Oujda 60000, Morocco; boussetta.reda@ump.ac.ma; 3 Laboratory of Innovation in Science, Technology and Education, CRMEF, Oujda 60000, Morocco; 4 Research Laboratory in Sciences and Techniques, ESEFA, Ibnou Zohr University, Agadir 80000, Morocco; 5 Grupo de Materia Condensada-UdeA, Instituto de Física, Facultad de Ciencias Exactas y Naturales, Universidad de Antioquia UdeA, Calle 70 No. 52-21, Medellín 050010, Colombia; cduque_echeverri@yahoo.es

**Keywords:** 2D-Curved nanostructure, electric field, magnetic field, transition energy, dipole matrix element, optical absorption, relative refractive index

## Abstract

Using the effective mass approximation and the finite difference method, we examined the linear, non-linear, and total optical absorption coefficients (OAC), as well as the relative refractive index coefficients (RIC) variations for an off-center shallow donor impurity in a 2D-curved electronic nanostructure subjected to external electric and magnetic fields. Our results reveal that the peak positions of the OAC and RIC are susceptible to the geometrical angles, the impurity position, and the strength of the applied electric and magnetic fields. In particular, the positions of the OAC and RIC peaks can be shifted towards blue or red by adjusting the geometric angle. In addition, the amplitudes of these peaks are influenced by the application of external fields and by the position of the impurity. This knowledge is essential for understanding and optimizing the optical characteristics of 2D-Curved nanostructure for advanced optoelectronic applications.

## 1. Introduction

Recent advances in nanofabrication have enabled the creation of 2D-curved nanostructures (2DCN), characterized by three-dimensional symmetry while confining electrons to two dimensions. Curved nanostructures with cylindrical geometry (controlled by three parameters: curvature radius, curvature angle and length) exhibit several unique physical properties compared to planar systems (controlled by two parameters: length and width), mainly due to the modification of the electronic structure and interactions induced by curvature [1,2,3]. By confining electrons to two dimensions at the atomic scale, curvature modifies the local density of states and wave functions of electrons. This modification significantly influences the electronic properties and linear optical absorption coefficient [3]. In curved 2DES, wave functions can localize near regions of high curvature, inducing variations in electron mobility and transport properties [4].

2DCN can be experimentally fabricated by strain-induced micro- and nanoscale patterning [5,6]. This process relies on the partial release of strain in a planar layer, thereby modifying the configuration of the crystal lattice and leading to significant changes in physical properties such as semiconductor bandgap energies [7] and charge carrier mobility [8]. Advanced techniques such as X-ray microdiffraction are used to characterize the distribution of deformations in these coiled tubes. Hosoda et al. [9] have fabricated and experimentally studied quantum well microtubes from GaAs, showing that these ultra-thin structures retain their quantum properties and that the optical emission efficiency is significantly improved after microtube formation. Unlike planar systems, these curved structures, such as spherical surfaces [10,11] and rolled multilayers [12,13,14], exhibit unique physical properties due to their curvature. The fabrication of 2DCN typically involves rolling strained semiconductor layers, followed by the removal of a sacrificial layer [5,6,15]. In these structures, the partial strain relaxation from the planar layer modifies the lattice configuration, resulting in changes to properties like semiconductor bandgap energies [16] and charge carrier mobility [8,17]. The introduced curvature intensifies confinement and resonance characteristics [18], enhancing the performance of high-precision photonic devices [19].

Research on the effects of donor impurities in these curved systems is still in its early stages. Impurities, whether intentionally introduced or due to fabrication defects, can significantly influence the electronic [20,21,22,23] and optical properties [24,25] of nanostructures. Understanding these effects is essential for optimizing and developing advanced devices based on these systems [18,26,27,28]. Recent studies have explored the effects of external influences on nanostructures. Hu et al. [29,30] explored the role of deformation on the properties of van der Waals materials, highlighting the impact of geometrical factors on optical responses. They have looked at van der Waals magnets embedded in polymer matrices, highlighting the influence of deformation and geometry on optoelectronic performance.

The linear and nonlinear optical properties of quantum dot (QD) systems have been extensively investigated both experimentally and theoretically [31,32,33]. Numerous studies have examined how external perturbations, such as electric and magnetic fields, influence these systems’ electronic and optical characteristics [34,35,36,37]. These perturbations are essential for tuning oscillator strength and peak resonance frequencies, allowing for precise control of optical responses. Previous research has explored the impact of electric fields on the electronic and optical properties of 2DCN. Pramjorn et al. [38] studied the binding energies of a donor impurity in a 2DCN under an electric field using the effective mass approximation and the finite difference method. They found that the binding energies increase with a smaller radius and can be controlled by the direction of the electric field. Belamkadem et al. [39] theoretically examined the surface properties of electrons in ultrathin spherical nanoflakes of QDs with centered or off-centered donor impurity. They used the finite difference method to analyze the energies and wave functions of the electrons. They observed that the ground state binding energy strongly depends on the geometry of the nanoflake and that the presence of an off-centered donor impurity allows for precise control of the binding energy, thus enhancing the functionality of these curved surface nanostructures. Recently, we examined the effects of surface curvature, electric fields, and impurity position on the electronic and linear optical absorption coefficients of hydrogenic donor impurity in 2D nanostructures [3]. Using the effective mass approximation and the finite difference method, we have found that these factors significantly influence the binding energy, transition energy, and optical characteristics.

The present research aims to further this work by investigating how electric and magnetic fields affect the linear, non-linear and total properties of optical absorption coefficients (OAC) as well as the relative refractive index change (RIC). To our knowledge, no comprehensive study has yet provided an analysis of the optical properties of curved 2DCN under external electric and magnetic fields in the presence of an off-center impurity. This research will theoretically examine these effects using the effective mass approximation, with energy levels determined by the finite difference method, considering the position of the donor impurity. The article is organized as follows: In Section 2, the theoretical framework outlines our study. The results and discussion are presented in Section 3, exploring the behavior of optical properties and examining the effects of external influences. The article concludes with the conclusions in Section 4, which highlights the main findings of our research.

## 2. Theoretical Framework

### 2.1. The Schrödinger Equation and Its Solution

**Figure 1:**
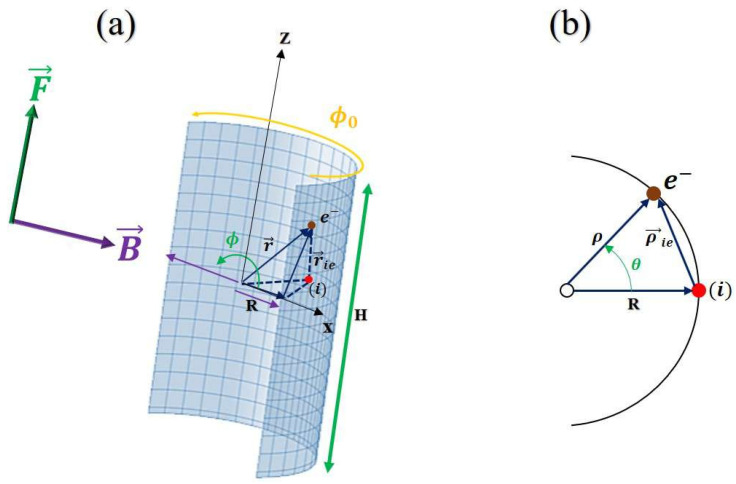
(**a**) Schematic representation of the cylindrical GaAs 2DCN. The dimensions of the 2DCN are defined by *H* for height, *R* for curvature radius, and $\phi_{0}$ for length. The applied electric field is oriented in the *z*-direction, while the magnetic field is aligned in the *x*-direction. The projection of our system onto the *xy*-plane is shown in (**b**).

This study focuses on a 2D-Curved nanostructure, modeled as a cylindrical GaAs structure. The system is characterized by a radius *R*, an angle of curvature $\phi_{0}$, and a height *H*. It contains an off-center hydrogenic impurity located move azimuthally at position $\phi_{i}=f\left(\phi_{0}\right)$, as shown in Figure 1a. The 2DCN is subjected to two external perturbations: an electric field applied along the *z*-axis (0, 0, $F_{z}$) and a radially directed magnetic field ($B_{x}$, 0, 0) along the *x*-axis. We assume that the well is sufficiently narrow for the electron to be constrained to evolve only on a cylindrical surface of radius *R*. The Hamiltonian of a lateral surface of a cylinder can be simplified by considering certain approximations when the cylinder’s diameter *d* is much smaller than its radius *R*. By neglecting terms of the order d/R, the radial motion degrees of freedom can be adiabatically separated from those along the surface. Focusing on the lowest subband in the radial direction, the Hamiltonian for our system, considering the donor impurity and the influence of the external electric and magnetic fields, is given by: [3]:

(1) $$H_{D}=-\frac{\hbar^{2}}{2\;m_{e}^{*}}\left(\frac{1}{R^{2}}\frac{\partial^{2}}{\partial \phi^{2}}+\frac{\partial^{2}}{\partial z^{2}}\right)+\frac{i\;e\;\hbar }{m_{e}^{*}}\vec{A}\cdot \vec{\nabla }+\frac{e^{2}}{2\;m_{e}^{*}}{\vec{A}}^{2}+W_{i}\left(\phi ,z\right)+W_{C}\left(\phi ,z\right)-W_{D}+e\;\vec{F}\cdot \vec{r},$$

where, $m_{e}^{*}$ represents the electron effective mass, given by $m_{e}^{*}=0.067\;m_{0}$, where $m_{0}$ is the free electron mass. The term $\frac{i\;e\;\hbar }{m_{e}^{*}}\vec{A}\cdot \vec{\nabla }$ accounts for the kinetic energy of the electron in the presence of the magnetic field, while $\frac{e^{2}}{2\;m_{e}^{*}}{\vec{A}}^{2}$ represents the interaction of the magnetic field with the electron. The Coulomb potential due to the donor impurity is [22]:

(2) $$W_{i}\left(\phi ,z\right)=-\;\frac{e^{2}}{4\;\pi \;\epsilon_{0}\;\epsilon_{r}\sqrt{2\;R^{2}\;\left(1-\cos\left(\phi -\phi_{i}\right)\right)+{\left(z-z_{i}\right)}^{2}}}$$

where $\epsilon_{0}$ is the permittivity of free space, and $\epsilon_{r}$ is the relative permittivity. The coordinates $\phi_{i}$ and $z_{i}$ denote the impurity position, with $z_{i}=H/2$. The term $W_{D}$ signifies the geometric potential arising from the curvature of the cylindrical surface [14,40]:

(3) $$W_{D}=\frac{\nu_{R}\;\hbar^{2}}{8\;m_{e}^{*}\;R^{2}}\;,$$

where $\nu_{R}$ is a parameter that accounts for the effects of curvature and strain. For an unstrained system, $\nu_{R}=1$. However, in the presence of strain, $\nu_{R}$ can increase significantly, reflecting the impact of deformation on the system’s geometry [14]. The confinement potential $W_{C}\left(\phi ,z\right)$ is given by:

(4) $$W_{C}\left(\phi ,z\right)=\left\{\begin{array}{cc} 0 & \mathrm{if}\;0\leq \phi \leq \phi_{0}\;\mathrm{and}\;0\leq z\leq H\\ \infty & \mathrm{otherwise}. \end{array}\right.$$

The external electric field contributes to the Hamiltonian as follows:

(5) $$e\;\vec{F}\cdot \vec{r}=e\;F\;z\;.$$

The magnetic effects are incorporated into the Hamiltonian through the vector potential, $\vec{A}$. In cylindrical coordinates, the vector potential for a magnetic field applied along the *x*-axis is:

(6) $$\vec{A}=-\frac{1}{2}\;\vec{r}\times \vec{B}=\left(0,\frac{z\;B_{x}}{2},-\frac{y\;B_{x}}{2}\right).$$

The Hamiltonian, including the magnetic field effects, is expressed in a cylindrical quantum dot as follows:

(7) $$\begin{array}{c} H_{D}=-\frac{\hbar^{2}}{2\;m_{e}^{*}}\left(\frac{1}{R^{2}}\frac{\partial^{2}}{\partial \phi^{2}}+\frac{\partial^{2}}{\partial z^{2}}\right)+\frac{i\;e\;\hbar }{m_{e}^{*}}\left(z\;\sin\phi \;\frac{\partial }{\partial \phi }+\frac{1}{R}\;\cos\phi \;\frac{\partial }{\partial \phi }-R\;\sin\phi \right)\\ +\frac{e^{2}}{8\;m_{e}^{*}}\left(z^{2}+R^{2}\;\sin^{2}\phi \right)B_{x}^{2}-\frac{\hbar^{2}}{8\;m_{e}^{*}\;R^{2}}-\frac{e^{2}}{4\;\pi \;\epsilon_{0}\;\epsilon_{r}\;\sqrt{2\;R^{2}\;\left(1-\cos\left(\phi -\phi_{i}\right)\right)+{\left(z-z_{i}\right)}^{2}}}\\ +W_{C}\left(\phi ,z\right)+e\;F\;z\; \end{array}$$

We replace continuous derivatives with finite differences on a discrete grid to discretize the provided Hamiltonian using the finite difference method (FDM). For the grid points in (ϕ,z), denote ψ(ϕ,z) as $\psi_{k,l}$, where *k* and *l* index the grid points along ϕ and *z*, respectively. We employ a finite difference technique to discretize the equations on a 2D grid comprising $T_{\phi }\times P_{z}$ grid points. The domain $\left[0,\phi_{0}\right]$ is partitioned into $\left(T_{\phi }+1\right)$ segments, and [0,z] is segmented into $\left(P_{z}+1\right)$ parts. The indices $1\leq k\leq T_{\phi }$ and $1\leq l\leq P_{z}$ correspond to integer values representing the positions of the grid points along the 2D QD, characterized by the coordinates ϕ and *z*. The intervals between these points are Δϕ and Δz, where $\phi_{0}=k\;\Delta \phi$ and H=lΔz. To approximate the second-order partial derivatives $\frac{\partial^{2}}{\partial \phi^{2}}$ and $\frac{\partial^{2}}{\partial z^{2}}$, the first derivatives $\frac{\partial }{\partial \phi }\psi_{D}\left(\phi ,\theta ,\phi \right)$, we use the second-order central difference method. The discretized equation at a grid point (k,l) is formulated as follows:

(8) $$\begin{array}{c} \frac{\partial^{2}\psi }{\partial \phi^{2}}\approx \frac{\psi_{k+1,l}-2\;\psi_{k,l}+\psi_{k-1,l}}{{\left(\Delta \phi \right)}^{2}}\\ \frac{\partial \psi }{\partial \phi }\approx \frac{\psi_{k+1,l}-\psi_{k-1,l}}{2\;\Delta \phi }\\ \frac{\partial^{2}\psi }{\partial z^{2}}\approx \frac{\psi_{k,l+1}-2\;\psi_{k,l}+\psi_{k,l-1}}{{\left(\Delta z\right)}^{2}}\;. \end{array}$$

At grid point (k,l), the discretized form of the Hamiltonian equation becomes:

(9) $$\begin{array}{c} H_{D}=\left[\begin{array}{c} -\frac{\hbar^{2}}{2m_{e}^{*}R^{2}{\left(\Delta \phi \right)}^{2}}-\frac{\hbar^{2}}{2m_{e}^{*}{\left(\Delta z\right)}^{2}}+\frac{e^{2}}{8m_{e}^{*}}\left(z^{2}+R^{2}\sin^{2}\Delta \phi \right)B_{x}^{2}-\frac{\hbar^{2}}{8m_{e}^{*}R^{2}}+W_{c}\left(\phi_{k},z_{l}\right)+eF\Delta z\\ -\frac{e^{2}}{4\pi \epsilon_{0}\epsilon_{r}\sqrt{2R^{2}\left(1-\cos\left(\Delta \phi -\Delta \phi_{i}\right)\right)+{\left(\Delta z-\Delta z_{i}\right)}^{2}}} \end{array}\right]\psi_{k,l}\\ +\left[-\frac{\hbar^{2}}{2m_{e}^{*}R^{2}{\left(\Delta \phi \right)}^{2}}+\frac{ie\hbar }{m_{e}^{*}}\frac{\Delta z\sin\Delta \phi }{2\Delta \phi }+\frac{ie\hbar }{m_{e}^{*}R}\frac{\cos\Delta \phi }{2\Delta \phi }\right]\psi_{k+1,l}\\ +\left[-\frac{\hbar^{2}}{2m_{e}^{*}R^{2}{\left(\Delta \phi \right)}^{2}}-\frac{ie\hbar }{m_{e}^{*}}\frac{\Delta z\sin\Delta \phi }{2\Delta \phi }-\frac{ie\hbar }{m_{e}^{*}R}\frac{\cos\Delta \phi }{2\Delta \phi }\right]\psi_{k-1,l}+\left[-\frac{\hbar^{2}}{2m_{e}^{*}{\left(\Delta z\right)}^{2}}\right]\psi_{k,l+1}\\ +\left[-\frac{\hbar^{2}}{2m_{e}^{*}{\left(\Delta z\right)}^{2}}\right]\psi_{k,l-1}. \end{array}$$

$T_{\phi }$ and $P_{z}$: Number of grid points along the ϕ and *z* axes, respectively. Here, $\Delta \phi =\phi_{0}/\left(T_{\phi }+1\right)$ denotes the pitch, representing the distance between two consecutive grid nodes in the $\vec{e_{\phi }}$ direction. Similarly, $\Delta z=H/\left(P_{z}+1\right)$ is the pitch, indicating the angle between two consecutive grid points in the $\vec{e_{z}}$ direction.

### 2.2. Optical Properties

The photon absorption in a quantum system involves an electron moving from a lower to a higher energy state by absorbing a photon. This transition occurs if the photon energy, ℏω, is at least equal to the energy difference between the ground state and the first excited state, $E_{1}-E_{0}$, where $E_{0}$ and $E_{1}$ denote the energies of the ground and first excited states, respectively. The optical absorption can be categorized into linear, third-order nonlinear, and total between these levels. The formula for the linear optical absorption coefficients is expressed as [41,42]:

(10) $$\alpha^{\left(1\right)}\left(\omega \right)=\sqrt{\frac{\mu }{\varepsilon_{r}}}\frac{\sigma_{d}\;\hbar \;\omega \;{\left|M_{01}\right|}^{2}\;\Gamma_{01}}{{\left(E_{01}-\hbar \;\omega \right)}^{2}+{\left(\hbar \;\Gamma_{01}\right)}^{2}}\;.$$

The following formula gives the expression for the third-order non-linear OAC [43,44]:

(11) $$\alpha^{\left(3\right)}\left(\omega ,I\right)=-\omega \sqrt{\frac{\mu }{\varepsilon }}\left(\frac{2\;I}{\epsilon_{0}\;n_{r}\;c}\right)\frac{\sigma_{d}\;\hbar \;\Gamma_{01}\;{\left|M_{01}\right|}^{4}}{{\left[{\left(E_{01}-\hbar \;\omega \right)}^{2}+{\left(\hbar \;\Gamma_{01}\right)}^{2}\right]}^{2}}\;.$$

Using Equations (10) and (11), one can express the total absorption coefficient as:

(12) $$\begin{array}{cc} \; & \alpha \left(\omega ,I\right)=\alpha^{\left(1\right)}\left(\omega \right)+\alpha^{\left(3\right)}\left(\omega ,I\right) \end{array}$$

In this context, $\sigma_{d}$ represents the electron density, $\varepsilon_{r}$ is the relative permittivity, and $\Gamma_{01}$ is the relaxation rate between the states |0〉 and |1〉, with $\Gamma_{01}$ defined as $\Gamma_{01}=\frac{1}{\tau_{01}}$. For this study, $\tau_{01}=0.14\;\mathrm{ps}$.

The energy difference between $E_{0}$ and $E_{1}$ is denoted by $E_{01}$. The dipole transition matrix element $M_{01}$, which indicates the strength of the transition between these levels, is given by [45]:

(13) $$M_{01}=\langle \psi_{0}|e\;z|\psi_{1}\rangle ,$$

where $\psi_{0}$ and $\psi_{1}$ are the wave functions of the ground and the first excited states, respectively.

The linear RIC shift of order one of the transition between these states is [46,47]:

(14) $$\frac{\Delta n_{r}^{\left(1\right)}\left(\omega \right)}{n_{r}}=\frac{\sigma_{d}{\left|M_{01}\right|}^{2}}{2\;n_{r}^{2}\;\varepsilon_{0}}\frac{\left(E_{01}-\hbar \;\omega \right)}{{\left(E_{01}-\hbar \;\omega \right)}^{2}+{\left(\hbar \;\Gamma_{01}\right)}^{2}}\;.$$

The expression for the nonlinear RIC is [48,49]:

(15) $$\frac{\Delta n_{r}^{\left(3\right)}\left(\omega ,I\right)}{n_{r}}=-\frac{\mu \;I\;c\;\sigma_{d}\;{\left|M_{01}\right|}^{4}}{n_{r}^{3}\;\varepsilon_{0}}\frac{\left(E_{01}-\hbar \;\omega \right)}{{\left[{\left(E_{01}-\hbar \;\omega \right)}^{2}+{\left(\hbar \;\Gamma_{01}\right)}^{2}\right]}^{2}}\;.$$

The total RIC shift is:

(16) $$\frac{\Delta n_{r}\left(\omega ,I\right)}{n_{r}}=\frac{\Delta n_{r}^{\left(1\right)}\left(\omega \right)}{n_{r}}+\frac{\Delta n_{r}^{\left(3\right)}\left(\omega ,I\right)}{n_{r}}\;.$$

## 3. Results and Discussion

Based on the theoretical model outlined above, we will investigate how the angle of the 2DCN, the applied electric field, the applied magnetic field, and the position of the donor impurity affect the electronic spectrum of the system. The position of the azimuthal angle of the donor impurity atom is defined as follows: $\phi_{i}=0$ (at the edge), $\phi_{i}=\phi_{0}/2$ (in the middle) and $\phi_{i}=\phi_{0}/4$ (between the middle and the edge).

**Figure 2:**
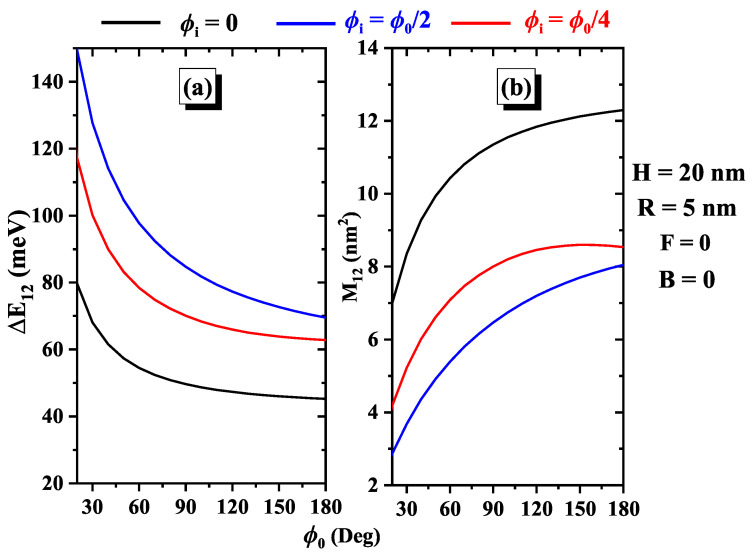
Variations of the transition energy (**a**) and the dipole matrix element (**b**) are shown as a function of the geometric angle for three impurity positions: $\phi_{i}=0$, $\phi_{i}=\phi_{0}/4$, and $\phi_{i}=\phi_{0}/2$. Results are for H=20 nm and R=5 nm, in the absence of an electric field and magnetic field.

Figure 2 depicts the relationship between the electron transition energy, $\Delta E_{12}$, and the dipole matrix element, $|M_{12}|$, concerning the geometric angle $\phi_{0}$, shown in Figure 2a and 2b, respectively, for different donor impurity positions. Panel (a) reveals that the transition energy decreases as the geometric angle increases. This decrease corresponds to a redshift, indicating that transitions occur at lower energies as $\phi_{0}$ becomes larger. From panel (b) it is evident that $|M_{12}|$ increases steadily with the orientation of the geometric angles, reaching a maximum plateau at the critical value of $\phi_{0}=140^{\circ }$. This observation aligns with previous results, indicating stronger wave function overlap due to the enhanced localization of charge carriers at this specific angle. Notably, $\Delta E_{12}$ reaches a maximum value when the impurity is positioned at $\phi_{i}=\phi_{0}/2$ (blue color curve), whereas $|M_{12}|$ reaches a minimum at the same position. The physical reason for this behavior is that higher structural confinement leads to reduced wavefunction overlap. These opposite trends suggest an inverse relationship between $\Delta E_{12}$ and $|M_{12}|$ as a function of $\phi_{0}$. The unique behavior at $\phi_{i}=\phi_{0}/2$ indicates that this specific position creates a distinct configuration or interaction within the nanostructure, requiring the highest transition energy while minimizing the matrix element interactions. Understanding these relationships is essential for tuning material properties, as positioning impurity spatially at $\phi_{i}=\phi_{0}/2$ could optimize the transition energy, whereas adjusting $\phi_{0}$ could enhance the matrix element interactions, offering valuable insights for targeted applications and further studies. The behaviors of the $\Delta E_{12}$ and the modulus of the matrix element $|M_{12}|$ are closely related.

**Figure 3:**
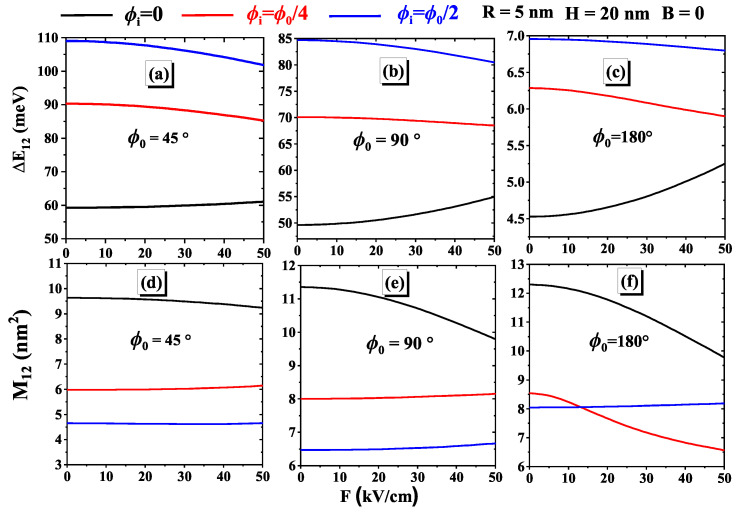
Variations of the transition energy (**a**–**c**) and the matrix element (**d**–**f**) as a function of the electric field for three impurity positions. Calculations are for H=20 nm and R=5 nm.

In Figure 3a–c display the transition energy $\Delta E_{12}$ as a function of the applied electric field for different geometric angles $\phi_{0}=45^{\circ }$, $\phi_{0}=90^{\circ }$, and $\phi_{0}=180^{\circ }$, respectively. Three positions of the impurity have been considered in each figure. Figure 3d–f illustrate the dipole matrix element $|M_{12}|$ under the same conditions. Generally, $\Delta E_{12}$ decreases with increasing the electric field due to the localization of the electronic wave function. An exception occurs when the impurity is at $\phi_{i}=0^{\circ }$, where $\Delta E_{12}$ increases due to strong electrostatic interactions leading to significant confinement. Conversely, $|M_{12}|$ tends to increase with the electric field, except when the impurity is at $\phi_{i}=0^{\circ }$, where it decreases gradually. These results are consistent with previous findings, indicating stronger wave function overlap due to increased localization of charge carriers. Moreover, $\Delta E_{12}$ reaches a maximum value when the impurity is positioned at $\phi_{i}=\phi_{0}/2$. This can be attributed to the fact that the ground state and the first excited states become more localized around the impurity as it approaches the center of the curved system. Meanwhile, $|M_{12}|$ reaches a minimum at this same angle. Except for $\phi_{0}=180^{\circ }$, a significant change in the behavior of $|M_{12}|$ is observed before and after F=15kV/cm. Specifically, for $\phi_{i}=\phi_{0}/2$, the value of $|M_{12}|$ is lower than that obtained for $\phi_{i}=\phi_{0}/4$, while the opposite behavior is observed when *F* exceeds 15kV/cm. These results show that the coupling between the electric field, the geometric angle, and the impurity position plays an essential role in the variation of $\Delta E_{12}$ and $|M_{12}|$. It is essential to understand these trends to tailor material properties, as adjusting the impurity positions and the intensity of the electric field can optimize transition energies and dipole matrix elements. This provides valuable insights for material design and further research [50,51].

**Figure 4:**
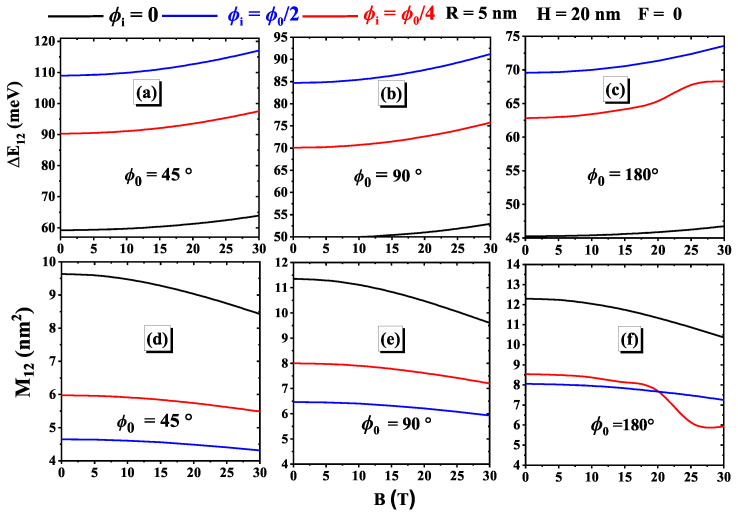
Variations of the transition energy (**a**–**c**) and the dipole matrix element (**d**–**f**) as a function of the magnetic field for three impurity positions and three geometric angles: (**a**) $\phi_{0}=45^{\circ }$, (**b**) $\phi_{0}=90^{\circ }$, and (**c**) $\phi_{0}=180^{\circ }$. These results are obtained for H=20 nm and R=5 nm.

Figure 4 depicts the transition energies and dipole matrix elements as a function of the magnetic field strength for fixed parameters, as mentioned in the previous figure. We observe that $\Delta E_{12}$ increases with the applied magnetic field, indicating that a higher magnetic field requires more energy for transitions, potentially due to enhanced electronic interactions and confinement effects. In contrast, $|M_{12}|$ decreases with increasing magnetic field, suggesting that the magnetic field weakens the interaction and overlap of matrix elements. Moreover, both $\Delta E_{12}$ and $|M_{12}|$ exhibit distinct behavior at the impurity position $\phi_{i}=\phi_{0}/2$ (as seen earlier) due to the tunnel effect caused by strong confinement induced by the magnetic field strength. Additionally, $\Delta E_{12}$ reaches a maximum, highlighting a peak in the required transition energy, while $|M_{12}|$ hits a minimum, indicating reduced dipole matrix element interactions at this angle. These trends reveal an inverse relationship between $\Delta E_{12}$ and $|M_{12}|$ in response to the magnetic field and underscore the significance of impurity positioning in determining the system behavior. Such insights are essential for designing materials with tailored properties, as adjusting impurity positions and magnetic field strengths can optimize transition energies and dipole matrix element interactions, informing both theoretical and practical advancements in material science. The variations in the transition energy $\Delta E_{12}$ and the modulus of the matrix element $|M_{12}|$ are closely correlated and agree well with the results observed in this study [52].

**Figure 5:**
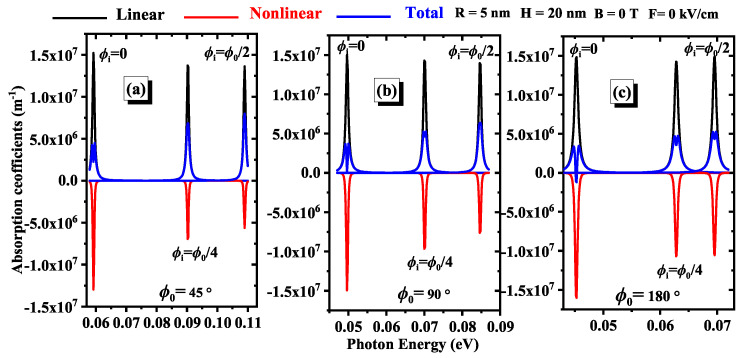
Variations of linear, nonlinear, and total optical absorption coefficients as a function of photon energy are analyzed for three geometric angles: (**a**) $\phi_{0}=45^{\circ }$, (**b**) $\phi_{0}=90^{\circ }$, and (**c**) $\phi_{0}=180^{\circ }$. The parameters R=5 nm and H=20 nm are fixed, and we consider the absence of electric and magnetic fields. Each figure displays results for various impurity positions.

In Figure 5a ($\phi_{0}=45^{\circ }$), Figure 5b ($\phi_{0}=90^{\circ }$), and Figure 5c ($\phi_{0}=180^{\circ }$) illustrate the effect of impurity position on the nonlinear optical absorption coefficients. As the impurity moves from $\phi_{i}=0^{\circ }$ to $\phi_{i}=\phi_{0}/2$, there is a noticeable red-shift in the peak of the AC, indicating that the peak shifts to higher energy levels. This red shift suggests that the required energy for nonlinear optical AC increases with the changing impurity position. The peak shift due to the impurity is identical to that observed in [38]. Additionally, the maximum value of the peak increases as the impurity position changes in the same direction, highlighting an enhancement in the OAC at higher energies. This effect is most pronounced at a geometric angle of $\phi_{0}=45^{\circ }$ (Panel (a)), where the changes in the absorption properties due to the impurity position are more significant. This pronounced effect at $\phi_{0}=45^{\circ }$ implies that the interaction between the electronic states and the impurity is particularly sensitive at this angle, leading to more substantial shifts and increases in the absorption peak. Understanding these trends is essential for designing materials with optimized nonlinear optical properties, as it provides insights into how impurity positioning can be strategically used to tailor the absorption characteristics for specific applications in photonics and optoelectronics [53].

**Figure 6:**
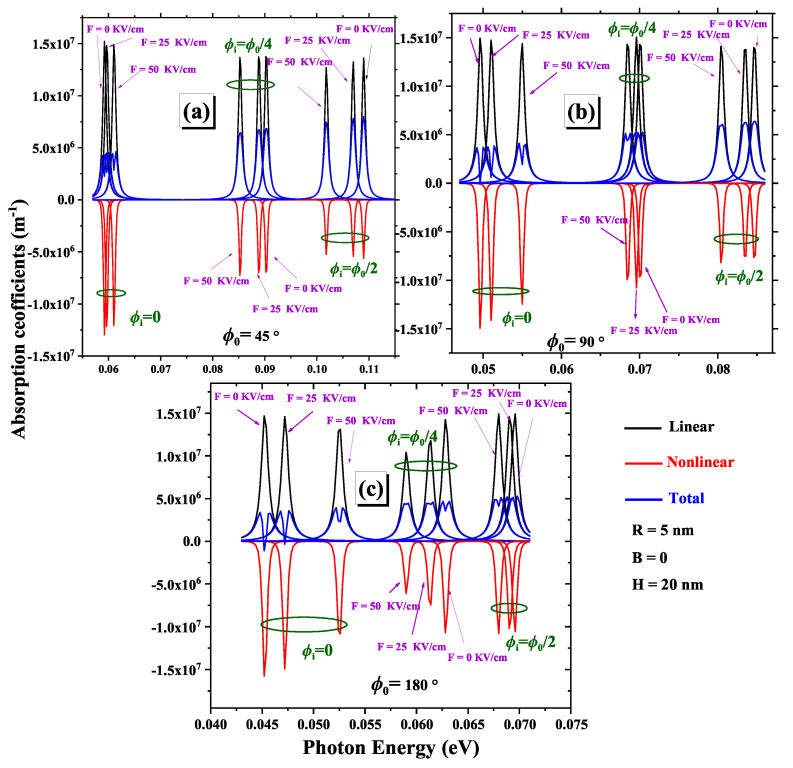
Variations of linear, nonlinear, and total optical absorption coefficients as a function of photon energy are analyzed for three geometric angles: (**a**) $\phi_{0}=45^{\circ }$, (**b**) $\phi_{0}=90^{\circ }$, and (**c**) $\phi_{0}=180^{\circ }$. The parameters R=5 nm and H=20 nm are fixed and the results are presented for various impurity positions and different electric field intensities.

This section provides a physical discussion that highlights the significance of external perturbations. In Figure 6, we explore how variations in the linear, nonlinear, and total OAC change with photon energy, considering different impurity positions $\phi_{i}$, electric field intensities, and geometric angles $\phi_{0}$. An increase in the electric field and a shift of the impurity towards the center result in a redshift of the absorption peaks for $\phi_{i}=0$ and a blueshift for $\phi_{i}=\phi_{0}/4$ and $\phi_{0}/2$, regardless of the chosen geometric angle. This behavior reflects the complexity of interactions between the electric field, impurity position, and the intrinsic properties of the 2DCN. Moreover, the intensity of the absorption peaks may increase or decrease with the electric field, depending on the impurity’s position. Additionally, $\alpha^{\left(1\right)}\left(\omega \right)$ is positive, indicating direct photon absorption, while $\alpha^{\left(3\right)}\left(\omega \right)$ is negative, reducing α(ω). As shown in Figure 6, the intricate interplay between the electric field, impurity position, and geometric angle provides opportunities for designing and optimizing 2DCN with tailored optical properties, potentially leading to new advancements in nanophotonics [54].

**Figure 7:**
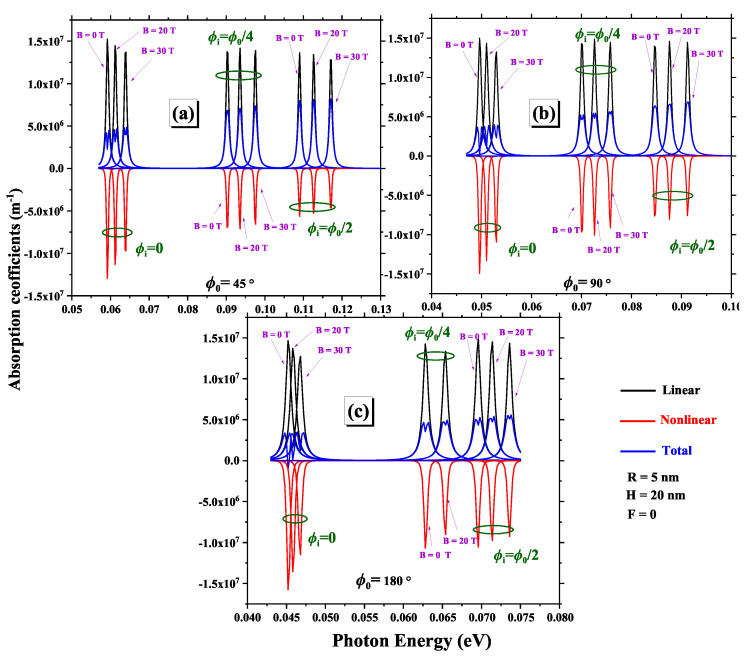
Variations of linear, nonlinear, and total optical absorption coefficients as a function of photon energy are analyzed for three geometric angles:(**a**) $\phi_{0}=45^{\circ }$, (**b**) $\phi_{0}=90^{\circ }$, and (**c**) $\phi_{0}=180^{\circ }$. The parameters R=5 nm and H=20 nm are fixed and the results are presented for various impurity positions and different magnetic field intensities.

In Figure 7, we examine how the linear, nonlinear, and total OAC vary with photon energy, considering different impurity positions $\phi_{i}$, magnetic field, and geometric angles $\phi_{0}$. The figure shows that increasing the magnetic field and moving the impurity towards the center of 2DCN causes absorption peaks to shift towards higher energies, a phenomenon accentuated by larger geometrical angles due to the increased interaction between curvature and magnetic field. In addition, at larger angles, curvature effects further modify electron confinement conditions, amplifying the impact of the applied magnetic field. In other words, the intensity of absorption peaks decreases with the magnetic field and when the impurity is moved towards the center. $\alpha^{\left(1\right)}\left(\omega \right)$ is always positive, and $\alpha^{\left(3\right)}\left(\omega \right)$ is negative, which reduces α(ω). The effect of the non-linear term, particularly noticeable at high optical intensities, becomes less pronounced with increasing magnetic field and impurity displacement towards the center, as magnetic confinement effects dominate, reducing the relative impact of non-linear interactions.

**Figure 8:**
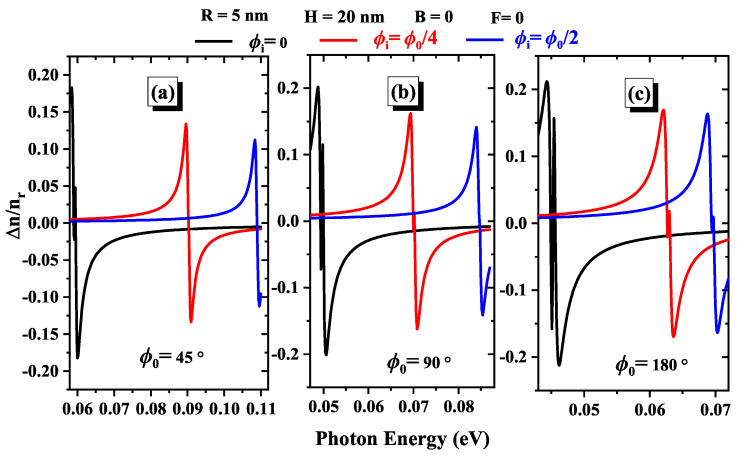
Variations of linear, nonlinear, and total RIC for three geometric angles: (**a**) $\phi_{0}=45^{\circ }$, (**b**) $\phi_{0}=90^{\circ }$, and (**c**) $\phi_{0}=180^{\circ }$. Each subfigure presents the results for various impurity positions.

In Figure 8a ($\phi_{0}=45^{\circ }$), Figure 8b ($\phi_{0}=90^{\circ }$), and Figure 8c ($\phi_{0}=180^{\circ }$) depict the effect of impurity position on the total RIC. As the impurity moves from $\phi_{i}=0$ to $\phi_{i}=\phi_{0}/2$, we observe a red shift in the peak of the total RIC, indicating that the peak shifts to higher energy levels. This shift suggests that the energy required to alter the RIC increases with the impurity position. Additionally, the maximum value of the peak decreases as the impurity position changes, indicating a reduction in the magnitude of the RIC at these higher energies. This effect is particularly pronounced at a geometric angle of $\phi_{0}=45^{\circ }$ (Panel (a)), where the impact of impurity positioning on the RIC is most significant. This pronounced effect at $\phi_{0}=45^{\circ }$ suggests that electronic interactions and state alignments are highly sensitive to impurity positions at this angle, resulting in more substantial shifts and decreases in the RIC peak. Understanding these trends is essential for designing materials with tailored optical properties, as it highlights how impurity positioning can be strategically utilized to optimize the nonlinear RIC for various applications in photonics and optoelectronics [55].

**Figure 9:**
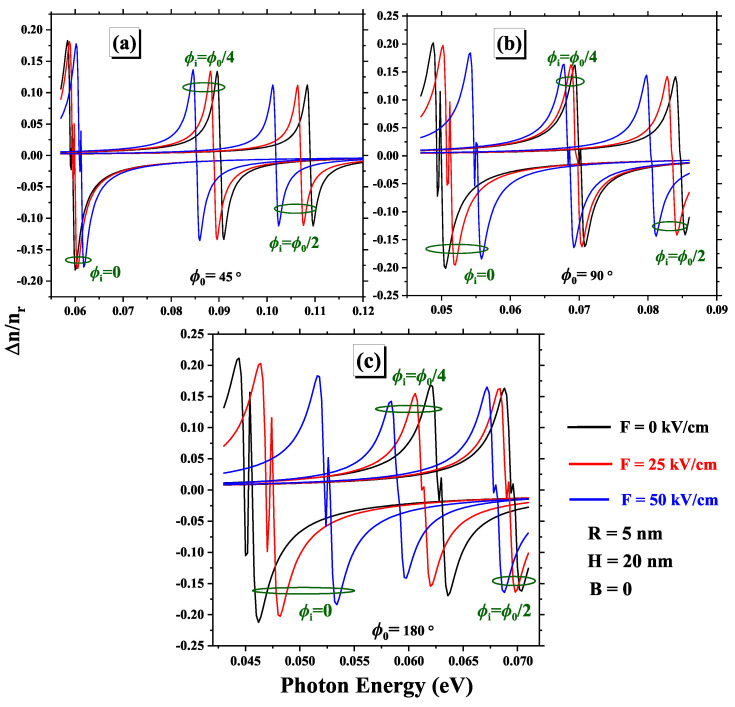
Variations of linear, nonlinear, and total RIC for three geometric angles: (**a**) $\phi_{0}=45^{\circ }$, (**b**) $\phi_{0}=90^{\circ }$, and (**c**) $\phi_{0}=180^{\circ }$. Each subfigure presents the results for various impurity positions and different electric field intensities.

Figure 9 shows how the total complex RIC depends on the energy of incident photons, impurity position $\phi_{i}$, and three different values of the electric field intensity for geometric angles of (a) $\phi_{0}=180^{\circ }$, (b) $\phi_{0}=90^{\circ }$, and (c) $\phi_{0}=45^{\circ }$. We observe that the absolute value of $\Delta n_{r}/n$ decreases as the electric field decreases, which is due to $\Delta n_{r}^{\left(3\right)}/n$ becoming a more significant term in the expansion, leading to an increase in the slope of the total RIC in the region where $\Delta n_{r}^{\left(1\right)}/n$ is zero. The peak amplitude of total RIC decreases with increasing electric field. whose behavior concerning the electric field has already been clarified. The shift of the impurity towards the edge of the 2DCN exhibits similar behavior, highlighting the influence of the impurity’s position on optical properties. In addition, a red shift of the RIC is observed as the impurity is located at $\phi_{i}=0^{\circ }$ and as the electric field increases. On the other hand, a blue shift is observed for impurities located at $\phi_{i}=45^{\circ }$ and $\phi_{i}=90^{\circ }$. This phenomenon is due to variations in $E_{12}$ with the electric field for different values of $\phi_{i}$, shown in Figure 3. In addition, the influence of *F* and $\phi_{i}$ on the RIC depends on $\phi_{0}$. At small $\phi_{0}$ angles, the curvature effect becomes dominant, significantly impacting the optical responses. Furthermore, previous studies [3] have shown that curved geometries introduce additional perturbations in quantum confinement. These studies highlight that at small $\phi_{0}$, curvature predominantly affects optical responses, whereas as $\phi_{0}$ increases, the properties of curved systems gradually converge towards those of 2D cylindrical systems. These findings confirm the relevance of curved nanostructures as a flexible platform for designing optoelectronic devices.

**Figure 10:**
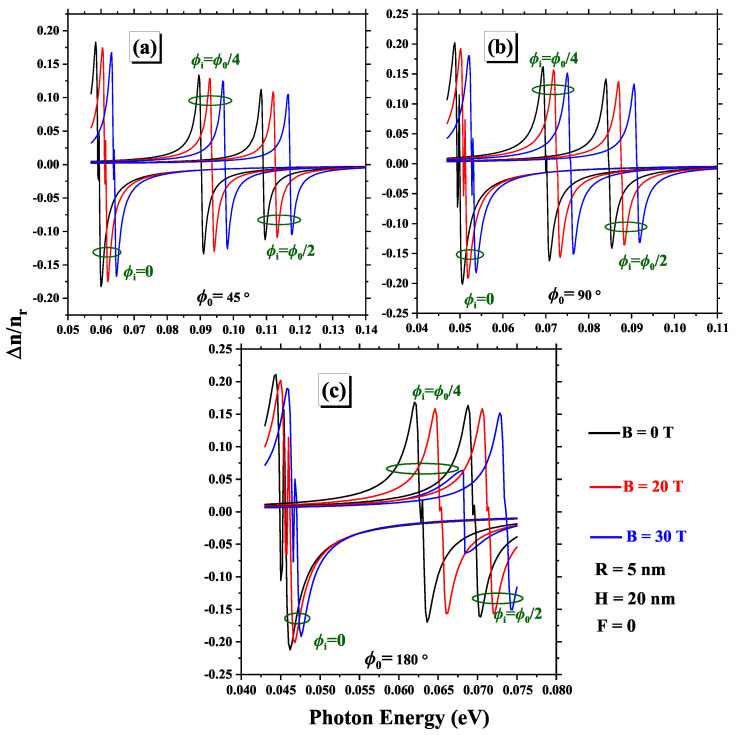
Variations of linear, nonlinear, and total RIC for three geometric angles: (**a**) $\phi_{0}=45^{\circ }$, (**b**) $\phi_{0}=90^{\circ }$, and (**c**) $\phi_{0}=180^{\circ }$. Each subfigure presents the results for various impurity positions and different magnetic field intensities.

The magnetic field plays an essential role in influencing the optical properties of 2DCN. When a magnetic field is applied, it modifies the behavior of electrons in the material by altering their movement and affecting electronic transitions. More specifically, the magnetic field can cause a separation of energy levels and influence the density of states available to electrons. These changes in energy levels have a direct impact on the way the system interacts with photons. For this reason, in Figure 10, we analyze how the total RIC behaves as the parameters of the 2DCN and the magnetic field change. In this figure, it is observed that the absolute value of $\Delta n_{r}^{\left(1\right)}/n$ decreases with decreasing magnetic field, as $\Delta n_{r}^{\left(3\right)}/n$ becomes a more dominant term in the expansion, which results in an increased slope of the total RIC where $\Delta n_{r}^{\left(1\right)}/n$ is zero. A similar trend is noted when the impurity is positioned closer to the edge of the 2DCN. Moreover, a blue shift of the RIC is observed, which becomes more pronounced with an increasing magnetic field, especially when the impurity moves toward the center. This shift can be attributed to the behavior of $E_{21}$ as a function of magnetic field strength for different values of $\phi_{i}$, as shown in Figure 4. Overall, these results highlight the significant interaction between the electric field, magnetic field, impurity location, and geometric angle in determining the optical properties of 2DCN.

Raman scattering is a crucial optical property that provides valuable insights into the behavior of 2D-curved nanostructures under various external influences. Its sensitivity to curvature effect, impurity positions, and external electric and magnetic fields makes it an essential tool for studying the interplay between structural effects and optical responses. In the context of our work, investigating Raman scattering alongside optical absorption coefficients and relative refractive index coefficients under the effects of geometric and external parameters would significantly enrich our findings. Recent advancements in this area [29,30], highlight its potential in enhancing the understanding of 2D-curved nanostructures. Therefore, as a natural extension of this study, we plan to investigate Raman scattering in future works to further explore and optimize the optical properties of these systems for advanced optoelectronic applications.

## 4. Conclusions

This study examines the impact of the azimuthal angle $\phi_{0}$ of 2D nanostructures, as well as the effects of the electric field, magnetic field, and impurity position, on the electronic spectrum of the system. The results show that the transition energy $\Delta E_{12}$ decreases with increasing $\phi_{0}$, indicating a redshift in electronic transitions, while the matrix element $|M_{12}|$ increases, reaching a maximum at $\phi_{0}=140^{\circ }$. Variations in the electric field generally lead to a decrease in $\Delta E_{12}$, except at $\phi_{0}=0^{\circ }$, and an increase in $|M_{12}|$, except at the same angle. The application of a magnetic field shifts the absorption peaks to higher energies and reduces the intensity of the matrix element. Additionally, the impurity position, along with the electric and magnetic fields, significantly influences the nonlinear optical absorption and the total refractive index coefficients. These findings demonstrate that applying electric and magnetic fields allows precise control over electronic transitions and associated optical responses. These phenomena are particularly pronounced due to the curved geometry of the studied structures, which alters the electronic density of states and wavefunctions, creating unique interactions between the applied fields and the optical properties of the nanostructures. This study provides deeper insights into the complex interplay between impurity, applied fields, and curved geometry, paving the way for novel applications in nanophotonics and optoelectronics.

## Data Availability

The data used to support the findings of this study are available from the corresponding author upon reasonable request.
